# Bleuler’s a or autism spectrum disorder in adults?

**DOI:** 10.1192/j.eurpsy.2021.1442

**Published:** 2021-08-13

**Authors:** J. Miranda, L. Silva, C. Almeida, I. Figueiredo, D. Machado, S. Fonseca

**Affiliations:** 1 Mental Health Department, Centro Hospitalar de Leiria, Leiria, Portugal; 2 Mental Health Department, Hospital Professor Doutor Fernando Fonseca, Lisboa (Amadora), Portugal

**Keywords:** autism, childhood onset schizophrenia, childhood psychosis, Early-onset schizophrenia

## Abstract

**Introduction:**

Nowadays we know that autism spectrum disorders (ASD) and Schizophrenic spectrum (SS) are different types of disorders in their etiology, symptoms and prognosis, but the clinical distinction is often difficult to make due to comorbidity and similar symptoms.

**Objectives:**

With this project, the authors intend to explore the differential diagnosis between ASD and SS specially when we talk about critical ages of onset.

**Methods:**

An analysis of articles searched on Pubmed (articles between 2010-2020) with the key words “adult autism”, “childhood onset schizophrenia”, “childhood psychosis”.

**Results:**

Early-onset schizophrenia (EOS) is defined as occurring before age 18 years. The condition share key diagnostic symptoms with adult-onset schizophrenia (AOS) but his prognoses and comorbidities differ. Autism spectrum disorder (ASD) is a common neurodevelopmental disorder characterized by difficulties since early childhood across reciprocal social communication and restricted interests and behaviors. ASD is a lifelong neurodevelopmental disorder, however there is a lack of answers and research for adults with ASD. There are shared aspects of odd thinking, rigid behaviors and impaired socialization in schizophrenia and ASD and COS seems to have a strong relationship with ASD, being comorbid in up to 50% of cases.

**Conclusions:**

Usually the evaluation of the developmental history of the person, prodrome and onset, its course and the presence of positive symptoms of schizophrenia is enough to help us find a diagnosis. Unfortunately, in some ages the conclusion is not so easy to find. However is essential to determine whether the clinical manifestations belong to the autistic spectrum, the schizophrenic or result from comorbidity.

